# Tricholemmal carcinoma of the head and neck region: A report of 15 cases

**DOI:** 10.3892/ol.2013.1726

**Published:** 2013-12-04

**Authors:** ZHIEN FENG, HAN-GUANG ZHU, LI ZHEN WANG, JIA-WEI ZHENG, WAN-TAO CHEN, ZHIYUAN ZHANG, WEI DONG, WEIGUO QU, YAN AN WANG

**Affiliations:** 1 Department of Oral and Maxillofacial Surgery, School of Stomatology, Peking University, Beijing 100081, P.R. China; 2 Department of Oral and Maxillofacial-Head and Neck Oncology, Ninth People’s Hospital, Shanghai Jiao Tong University School of Medicine, Shanghai 200011, P.R. China; 3 Department of Oral Pathology, Ninth People’s Hospital, Shanghai Jiao Tong University School of Medicine, Shanghai 200011, P.R. China; 4 Department of Oral and Maxillofacial Surgery, Dalian Stomatological Hospital, Dalian University, Dalian, Liaoning 116021, P.R. China

**Keywords:** tricholemmal carcinoma, clinicopathological features, treatment, prognosis, head and neck

## Abstract

Tricholemmal carcinoma is an extremely rare malignancy of the skin, and its biological behavior and management is controversial. The objective of the present study was to investigate the clinicopathological characteristics and management of tricholemmal carcinoma of the head and neck region. The study analyzed 15 patients with tricholemmal carcinoma. Demographic and clinical data were collected, and features associated with the management and prognosis of tricholemmal carcinoma were analyzed. Two of the 15 patients were lost to follow-up. The results showed that, during the follow-up period, 5 of the 13 available patients succumbed to the causes of recurrence (n=3), neck lymph node metastasis (n=1) and Parkinson’s disease (n=1). No patients developed distant metastasis. The disease-free survival (DFS) and overall survival (OS) were 31.1±7.8 and 32.9±7.4 months (mean ± SE), respectively, and the DFS and OS rates were 69.2 and 61.5%, respectively. In conclusion, the biological behavior of tricholemmal carcinoma is locoregionally aggressive. The recommended management for head and neck tricholemmal carcinoma is radical resection and neck dissection, and post-operative radiotherapy may be considered for high-risk patients.

## Introduction

Tricholemmal carcinoma, a rare malignant tumor, was first described in 1968 as tricholeptocarcinoma and occurred in 0.05% of the patients who underwent histopathological examinations (1). Currently, a definitive list of characteristic clinical signs of tricholemmal carcinoma is not available. Therefore, diagnosis is usually determined on the basis of the histological resemblance of the tumor to the outer root sheath, following the exclusion of other types of skin neoplasms (2), leading to a high misdiagnosis rate. Pathologically, tricholemmal carcinoma is characterized by an abundance of glycogen-rich, clear cells with foci of pillar-type keratinization, peripheral palisading of cells and subnuclear vacuolization (3). Additionally, the high mitotic rate appears to be a constant feature (4).

Despite the seemingly malignant cytological appearance of these lesions, clinical follow-up in the majority of cases shows that recurrence or metastasis is rare (2–4). Therefore, conservative surgical excision with the achievement of negative margins is considered the treatment of choice for these neoplasms. Tumor size and thickness are deemed to be the most important prognostic risk factors (5). Previously, it has also been shown that adjuvant radiotherapy is required for high-risk cases, when complete resection is impossible or if recurrence/metastasis has occurred (6–8).

The current study reports a series of 15 patients with tricholemmal carcinoma of the head and neck region, including one case example, with the focus on the clinicopathological characteristics and management of these lesions.

## Patients and methods

### Patient diagnosis

All patients provided written informed consent in accordance with the institutional guidelines of the Ninth People’s Hospital (Shanghai, China). Between April 1994 and September 2010, all 15 patients with tumors of the skin, pathologically diagnosed as tricholemmal carcinoma and treated at the Department of Oral and Maxillofacial Surgery, Ninth People’s Hospital, were enrolled into the current study. The pathological material from all the cases had been checked by two dermatopathologists who were blinded to the results. A consensus diagnosis of tricholemmal carcinoma was reached.

### Tissue preparation

For all patients, formalin-fixed, paraffin-embedded tissues were processed for routine microscopy, with histological sections being stained with hematoxylin and eosin (HE). Immunohistochemical (IHC) studies were performed on five patients. Paraffin-embedded tissue sections were incubated with the following selection of antibodies: Pan-cytokeratin (P-CK), keratin 15 (KRT15), vimentin (Vim), smooth muscle actin (SMA), S-100 protein, MelanA and human melanoma black (HMB)-45.

### Patient characteristics

Clinical information, which included age, gender, tumor site, size and treatment, was obtained from the medical records of the patients. The clinical course was followed by regular return visits and telephone interviews. The cutoff date for evaluation was June 1, 2012.

## Results

### Clinical observations

The clinical observations of the 15 patients are summarized in Table I. In total, there were 4 males and 11 females (male to female ratio, 1:2.75), with a mean age of 77.5 years (range, 24–92 years). In the majority of patients, the typical signs were a slow-growing exophytic or polypoid mass of recent onset and/or a surface ulcer combined with a bloody scab and keratotic nodule. Occasionally, patients also presented with pruritus or pain related to the mass.

Lesions were distributed on the face (n=11), temple (n=3) and nose (n=1). Multiple lesions were identified on the face of 1 of the 15 patients, and two other patients exhibited bilateral lesions (on the face and temples, respectively). The size of the tumors ranged between 1–13 cm in largest diameter. The lesions were most frequently misdiagnosed clinically as basal cell carcinoma. In total, 7 of the 15 cases underwent pre-operative biopsy or intraoperative frozen pathological testing. However, none of the primary cases were diagnosed as tricholemmal carcinoma prior to the results of the paraffin sections, reported following staining with HE and/or IHC.

All patients were treated by radical surgical excision, and neck dissection was performed for any patients with suspicion of lymph node (LN) metastasis (n=5). Ultimately, the neck LNs of two patients were positive, but all tumor margins were negative. The repair or reconstruction method for the defect, which was determined by the surgeons according to their clinical practice, included adjacent flap (n=11), pectoralis major myocutaneous flap (PMMF; n=2), free skin graft (n=2) and forearm flap (n=1). Post-operative radiotherapy was performed on two patients.

The follow-up results were available for 13 patients, since two patients were lost to follow-up. The median OS was 21 months (range, 7–99 months). Of the 13 patients, five (38.5%) succumbed to causes that included recurrence (n=3) and neck LN metastasis (n=1). One patient succumbed to a cause unrelated to the cancer (Parkinson’s disease). None of the patients developed distant metastases following treatment. The DFS and OS were 31.1±7.8 and 32.9±7.4 months (mean ± SE), respectively, and the DFS and OS rates were 69.2 and 61.5%, respectively.

### Pathological observations

All 15 cases showed an exophytic appearance and a whitish cut surface. Microscopically, the majority of cases showed an intraepithelial lesion that featured large cells with abundant clear cytoplasm. Specific cells showed intracytoplasmic keratohyalin granules and abrupt keratinization without a granular layer. The basal layer showed a tendency toward palisading and was associated with a prominent basement membrane. Nuclei were large and atypical; the degree of nuclear atypia and the high mitotic rate being sufficient to indicate malignancy, although no signs of dermal involvement were identified.

Although the majority of cases showed sharp borders, three cases showed an infiltrative neoplasm in which the epidermis and a large section of the dermis were replaced by large, cellular lobules. In addition, perineural invasion was observed in one case.

**Figure 1:**
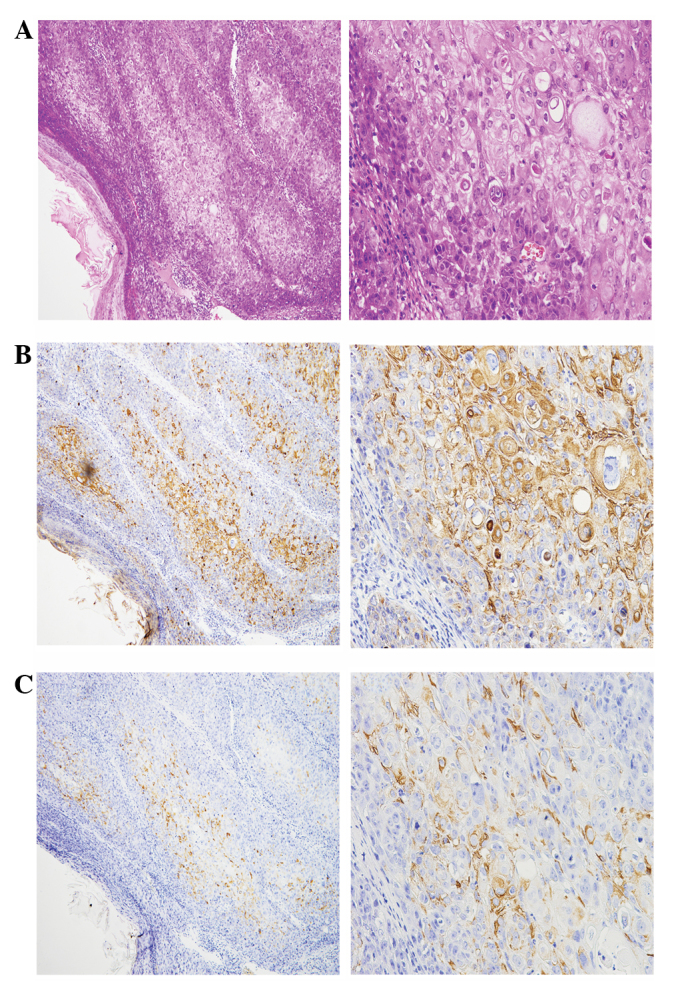
Typical histopathological appearance of head and neck tricholemmal carcinoma. (A) HE stain, (B) P-CK stain and (C) KRT15 stain. Magnification, left image ×200 and right image ×400. HE, hematoxylin and eosin; P-CK, pan-cytokeratin; KRT15, keratin 15.

The typical results of IHC staining were as follows: P-CK and KRT15 (a marker of outer root sheath originated tumors) (9) were positive, and Vim, S-100, SMA, MelanA and HMB-45 were negative. The HE and positive pathological features (P-CK and KRT15) are shown in Fig. 1.

### Typical case

**Figure 2:**
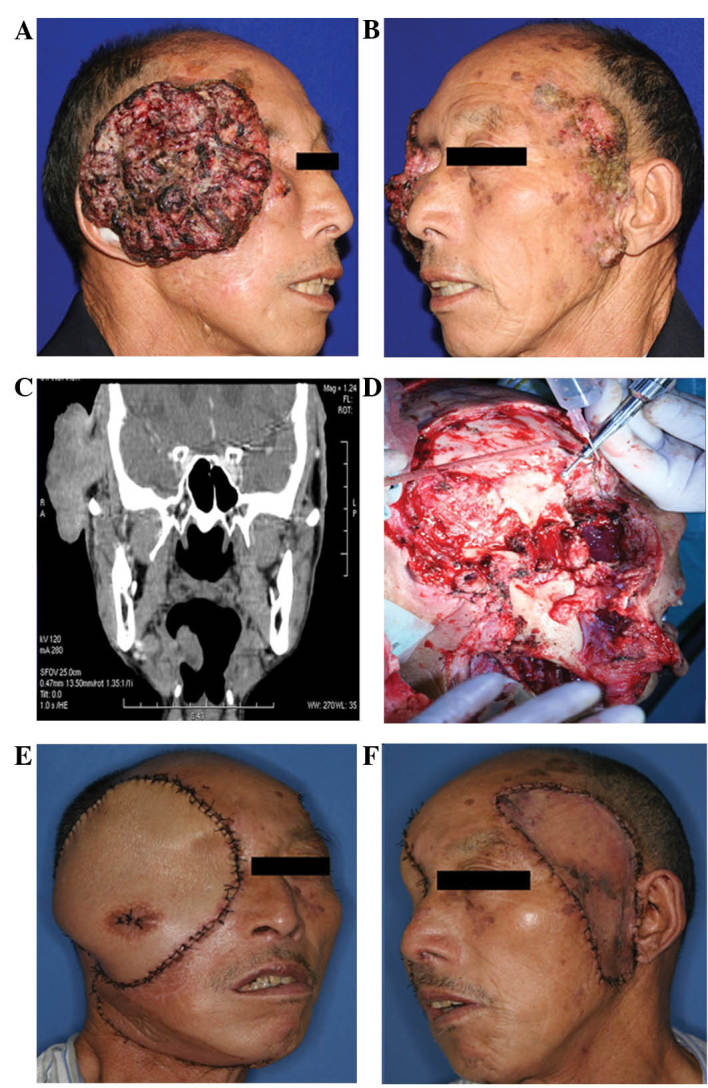
Clinical signs and treatment of a giant tricholemmal carcinoma with recurrence/multiple lesions. (A) Pre-operative image showing a giant lesion on the right temple. (B) Pre-operative image showing lesions on the left temple. (C) Pre-operative computed tomography image in the coronal plane. (D) Intraoperative image showing the appearance following radical resection of the lesions. (E) Post-operative image of the right-sided lesion taken 1 week later. (F) Post-operative image of the left-sided lesions taken 1 week later.

The clinical signs and treatment of a patient with a giant lesion with recurrence/multiple lesions are shown in Fig. 2 (case no. 7). This 69-year-old male presented with a 12×13-cm cutaneous tumor on the right temple and multiple cutaneous tumors on the left temple and left lower anterior tragus. Two years prior to the current admittance to the Department of Oral and Maxillofacial Surgery, Ninth People’s Hospital, the patient had undergone two surgeries at an alternative hospital in order to excise tumors from his right temple and orbit; the histological diagnosis being tricholemmal carcinoma. Only one year later, the tumor recurred again on the right temple. The size of the lesion on the right temple rapidly increased, whilst lesions on the left temple and maxillofacial region occurred in succession (Fig. 2A and 2B). The patient was transferred to the Department of Oral and Maxillofacial Surgery, Ninth People’s Hospital for further treatment.

A computed tomography scan of the cranial and maxillofacial region showed that the tumor was extensively infiltrating the soft tissues beneath the skin of the right temple. The right temporalis muscle and soft tissue of the lateral orbital margin were invaded (Fig. 2C). Therefore, a wide excision of the right-sided tumor was performed (Fig. 2D). A right radical neck dissection was also performed, and a PMMF was selected for reconstruction of the defect (Fig. 2E). The left-sided lesions were widely excised and the defect was repaired simultaneously with a free skin graft from the abdomen (Fig. 2F). The pathological diagnosis was recurrent tricholemmal carcinoma. All resection margins were negative, as were all neck LNs.

In view of the huge tumor size and thickness, post-operative radiotherapy (4–6 weeks after surgery) was strongly recommended for this patient. However, following an uneventful post-operative course, the patient declined to accept radiotherapy on schedule. Only 2 months later, the tumor recurred on the right temple and the patient succumbed to recurrence 7 months after surgery.

## Discussion

Tricholemmal carcinoma occurs predominantly on the scalps of elderly females, only occasionally arising outside the scalp. Lesion size is usually <2 cm, but sizes of ≤25 cm have been reported and 90% of the patients are Caucasian (10,11). The current study focused on the clinicopathological features and management of head and neck tricholemmal carcinoma in China. As previously described, tricholemmal carcinoma of the head and neck region has been most frequently misdiagnosed clinically as basal cell carcinoma (3).

In the 15 patients presented in the current study, the proportion of females was markedly higher compared with males (73.3 vs. 26.7%), which is consistent with the majority of previous reports (10), although the cause of this higher incidence rate among females is not clear. Recent preclinical and clinical observations have indicated that estrogen and its receptors may be involved in the development of skin cancer (12,13). Therefore, the hormonal changes of menopausal women may be an additional pathogenic factor in tricholemmal carcinoma of the head and neck region, in addition to sun exposure.

In the majority of previous studies, tricholemmal carcinoma was described as a solitary lesion (3,4,6), with multiple lesions being extremely rare. However, among the 15 patients of the present study, three patients (20.0%) had bilateral or multiple lesions. Frequent physical stimulation, which includes insolation and mechanical stimulation, is considered a key cause of carcinogenesis in the skin of the head and neck. These stimulatory factors cause various high-risk regions of the skin to transform into cancer, simultaneously or in succession. The results of the current study indicate that besides the main lesions in the head and neck, other plaques and abnormal skin must be closely followed up. Biopsies and preventative resections may be effective measures to decrease the occurrence of second cancers and improve cure rates.

The biological behavior of tricholemmal carcinoma remains controversial. A number of studies consider that these tumors have a favorable prognosis (3,4). Even for invasive cases and giant tumors, the reported rates of post-operative recurrence and metastasis have been extremely low following conservative, but thorough, excision (2,6). However, other studies consider the biological behavior of tricholemmal malignancy to be difficult to judge, reporting that although numerous patients have undergone local excision and post-operative radiotherapy, recurrence or LN metastasis is frequently found (14–16). An analysis of the prognosis in the present study showed three patients (20.0%) who exhibited neck LN metastasis and three patients who developed recurrence, which included one patient with repeated recurrences. As a result of recurrence or metastasis, four patients (30.8%) succumbed. In view of the evidence that none of the patients developed distant metastasis following treatment, we hypothesize that the biological behavior of head and neck tricholemmal carcinoma is more inclined to be locoregionally aggressive.

Further analysis showed that in three-quarters of the patients with recurrence or metastasis, the original tumor diameter was >3 cm, a result that is consistent with previous conclusions that tumor size and thickness are key prognostic factors for cutaneous carcinoma (5). Therefore, it is recommended that, for the management of tricholemmal carcinoma of large tumor size or thickness, wide excision and neck dissection must be performed. Post-operative radiotherapy for recurrent/LN metastasis patients may improve the locoregional control rate. For patients with small tumors, radical resection may lead to an improved prognosis, but close follow-up for possible neck LN metastasis is required.

The present study reported the clinicopathological features of head and neck tricholemmal carcinoma, which included multiple lesions, recurrence/metastasis and invasive growth, consistent with the overall biological behavior of this tumor being more aggressive. The recommended management for head and neck tricholemmal carcinoma must, therefore, be radical resection, with post-operative radiotherapy also performed for recurrent/metastatic lesions.

## Figures and Tables

**Table I:** Clinical observations of 15 patients with tricholemmal carcinoma of the head and neck region.

Case no.	Gender/age, years	Site	Longest diameter, cm	Recurrence	LN metastasis	Prognosis
1	F/78	Nose	1.5	No	Yes	Mortality
2	F/88	Temple	5.0	No	No	Mortality^b^
3	F/24	Face	3.0	No	No	Survival
4	F/83	Bilateral face	3.5	Yes	No	Mortality
5	F/91	Face	6.0	No	Yes^a^	Survival
6	M/87	Face	1.0	No	No	Survival
7	M/69	Bilateral temple	13.0	Yes	No	Mortality
8	F/83	Temple	2.0	No	No	Survival
9	M/81	Face	5.0	No	Yes^a^	Survival
10	F/90	Face	3.0	Yes	No	Mortality
11	F/92	Face	2.5	No	No	Survival
12	M/66	Face	3.0	Missing	Missing	Lost to follow-up
13	F/81	Face	3.0	No	No	Survival
14	F/57	Face (multiple lesions)	2.0	Missing	Missing	Lost to follow-up
15	F/92	Face	2.0	No	No	Survival

a Neck LN metastasis was apparent at diagnosis;

b cause of mortality was Parkinson’s disease.

LN, lymph node; F, female; M, male.
